# Bounded rationality alters the dynamics of paediatric immunization acceptance

**DOI:** 10.1038/srep10724

**Published:** 2015-06-02

**Authors:** Tamer Oraby, Chris T. Bauch

**Affiliations:** 1 Department of Mathematics, University of Texas - Pan American, Edinburg, Texas, USA; 2 Department of Applied Mathematics, University of Waterloo, Waterloo, Ontario, Canada

## Abstract

Interactions between disease dynamics and vaccinating behavior have been explored in many coupled behavior-disease models. Cognitive effects such as risk perception, framing, and subjective probabilities of adverse events can be important determinants of the vaccinating behaviour, and represent departures from the pure “rational” decision model that are often described as “bounded rationality”. However, the impact of such cognitive effects in the context of paediatric infectious disease vaccines has received relatively little attention. Here, we develop a disease-behavior model that accounts for bounded rationality through prospect theory. We analyze the model and compare its predictions to a reduced model that lacks bounded rationality. We find that, in general, introducing bounded rationality increases the dynamical richness of the model and makes it harder to eliminate a paediatric infectious disease. In contrast, in other cases, a low cost, highly efficacious vaccine can be refused, even when the rational decision model predicts acceptance. Injunctive social norms can prevent vaccine refusal, if vaccine acceptance is sufficiently high in the beginning of the vaccination campaign. Cognitive processes can have major impacts on the predictions of behaviour-disease models, and further study of such processes in the context of vaccination is thus warranted.

Processing imperfect information in limited time with bounded cognition affects individual decision-making. Risk aversion and ambiguity aversion render the rational decision model of classical utility theory more of a prescriptive model than a descriptive model^1^. Cognitive psychologists and behavioral economists have demonstrated such ramifications through numerous experiments, e.g.^1^,^2^, corroborated by the St. Petersburg, Allais, and Ellsburg paradoxes^3^. In the 1970s and 80s, H.A. Simon introduced the concept of bounded rationality to explain deviations from the rational decision model^4^,^5^,^6^. In the meantime, Kahneman and Tversky^7^ developed Prospect Theory (PT) through experimental psychology to explain and describe how individuals make decisions. According to PT, individuals measure their utility of each possible outcome based on how it is framed and on the perception/weight of the probabilities of its occurrence. Prospect theory has received significant attention in the decision-making community^1^,^2^,^8^, behavioral economics^9^,^10^,^11^, health^12^,^13^ and many other fields^14^,^15^,^16^,^17^.

Prospect theory attempts to explain how multitudinous everyday decisions are influenced by risk (mis)perception, range effect, loss aversion, diminishing sensitivity and framing effect. The decision whether or not to vaccinate should also be subject to these effects. While vaccination is a very efficient control measure for many paediatric infectious diseases^18^,^19^,^20^, some parents do not vaccinate their children. That appears to be due to the underestimation of disease risks and/or overestimation of vaccine risks, and vaccine-generated herd immunity^21^,^22^,^23^,^24^,^25^,^26^,^27^. According to the Health Belief Model (HBM), parents decide about childhood vaccination according to two types of variables: personal and social^22^,^28^,^29^,^30^,^31^. Personal variables include parental perceived risk of infection in terms of susceptibility and severity, perceived risk of vaccination, vaccine efficacy, vaccine cost, and its accessibility. For instance, low perceived vaccine efficacy can be detrimental to vaccination campaigns^32^. Social variables include injunctive social norms (group pressure) experienced through peers and health care providers. For instance, injunctive social norms affect vaccine uptake in different ways that depend on the initial size of the vaccinator group^33^. Other research has pointed to the ubiquitous role of social learning in vaccine decision-making, where individuals rely upon information provided by their broad social environment—peers, health care providers, and the media—to make their decisions^34^.

Many mathematical models of the interaction between vaccinating behavior and disease incidence predict that vaccination is not an effective method of disease elimination, because herd immunity makes vaccine refusal attractive to parents^25^,^32^,^33^,^35^,^36^,^37^,^38^,^39^,^40^,^41^,^42^. Refusal can emerge with or without a rational decision model. For example, behavioral economic models that incorporate bounded rationality can replicate the low acceptance rate of influenza vaccination in the United States^43^. Another behavioral economy framework found that subjective risks, efficacy, and cost of vaccination can also explain low acceptance rates^44^. The final size of epidemics and vaccine uptake were shown to be sensitive to strategic decisions towards vaccination in the case of imperfect information about disease risk in the presence of discount rate bias^45^, and when disease risk perception is based on partial recall of historical prevalence^46^. A similar conclusion is reached in the case where decisions are made to reduce contact rates, when disease risk perception depends on recalling the number of symptomatic cases over a certain past period of time^47^. Moreover, the clustering of opinions on networks itself (i.e. without feedback from disease dynamics) can also affect the herd immunity threshold in ways that make it harder to eliminate infection^48^. In contrast, other approaches observe that pediatric vaccine coverage is often higher than predicted by the conventional free-rider framework, and show that adding injunctive social norms or public health information to models can reconcile the models to this observation^33^,^41^. These and other “behavior-disease” models have begun moving away from the rational decision model in various ways^25^,^32^,^33^,^35^,^36^,^37^,^38^,^39^,^40^,^41^,^42^,^49^.

In this paper, we incorporate prospect theory into a behavior-disease model to investigate the dynamical behavior of parental acceptance of paediatric infectious disease vaccines under the bounded rationality paradigm. To our knowledge, this is the first paper that incorporates prospect theory into a behaviour-disease modelling framework that also includes parameters governing costs, effecacy and social norms. It thus allows us to capture how changes in individual cognitive processes described by prospect theory can affect population-level vaccine coverage and disease dynamics. Also, in contrast to how injunctive social norms are modelled in^33^, we allow injunctive social norms (group pressure) to differ between vaccinator and non-vaccinator groups, since in reality those two groups might impose pressures of different magnitudes. We study the dynamical behavior of the model and identify different regions with different long-term states, comparing them to outcomes in a special case of the model that lacks bounded rationality (i.e. a model that is closer to a rational decision model). We show how changes in the perceived risks of vaccine and disease can translate into different equilibria. Vaccine efficacy^32^ and cost of vaccination are also shown to affect the equilibrium at the same levels of risk perceptions. After analyzing the model to identify its dynamical regimes, we will then use some published parameter values to investigate the effect of the new parameters on the dynamical behavior of vaccine uptake/acceptance.

## Methods

### Modeling Decision-Making via Prospect Theory

A decision made according to prospect theory starts by editing the prospects via combining, segregating, canceling and simplifying the prospects^14^. A prospect Φ = (*z*_1_,*p*_1_; *z*_2_,*p*_2_; …; *z*_*k*_,*p*_*k*_), given after the editing phase, is defined by possible outcomes *z*_*i*_ (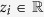) and their corresponding probabilities *p*_*i*_ (0 < *p*_*i*_ < 1). A prospect Φ = (*z*_1_,*p*_1_; *z*_2_,*p*_2_; …; *z*_*k*_,*p*_*k*_) has a utility given by


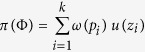


c.f.^50^. (Specifications of the two functions *ω* and *u* are given below.) However, in the case of a prospect of only two possible outcomes with the same sign, an adjustment is needed; that is, if Φ = (*z*_1_,*p*_1_; *z*_2_,*p*_2_) with *p*_1_ + *p*_2_ = 1 and either 0 < *z*_1_ < *z*_2_ or *z*_2_ < *z*_1_ < 0, then the utility would be given by *π*(Φ) = *u*(*z*_1_) + *ω*(*p*_2_)[*u*(*z*_2_) − *u*(*z*_1_)].

The decision-maker prefers a prospect Φ_1_ to another prospect Φ_2_ if *π*(Φ_1_) > *π*(Φ_2_) and is indifferent if *π*(Φ_1_) = *π*(Φ_2_). Prospect theory, however, violates the stochastic dominance axiom, because prospect Φ_1_ may be preferred to Φ_2_, even though outputs of prospect Φ_2_ stochastically dominate prospect Φ_1_ c.f.^50^. That violation was fixed later in cumulative prospect theory (CPT)^2^, but we use here the classical prospect theory as a tractable approximation to CPT (see Appendix III).

**Figure 1:**
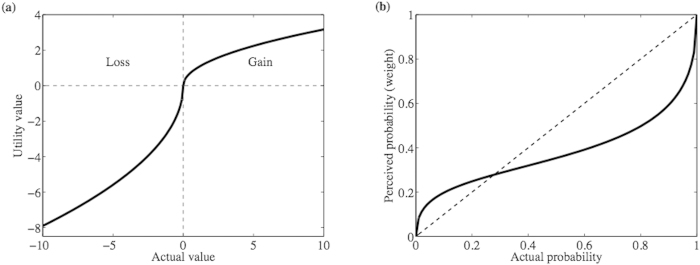
Utility function and probability perception/weight. (**a**) The gain and loss utilities of the objective/actual value measured with respect to a reference point. The plot corresponds to the parameters *α* = .5 and *λ* = 2.5. (**b**) The perception/weighting function *ω* of actual probabilities at parameter value *η* = .5, see equation (1).

The subjective utility function is defined by *u*(*x*) = *u*_*G*_(*x*)*I*_+_(*x*) + *u*_*L*_(*x*)(1 − *I*_+_(*x*)) where *I*_+_(*x*) is the indicator function that equals one if *x* is positive and zero otherwise, *u*_*G*_(*x*) = *x*^*α*^, and *u*_*L*_(*x*) = −λ(−*x*)^*α*^ such that 1 > *α* > 0 and λ > 1 is the loss aversion index^2^,^14^. The concavity of *u*_*G*_ and convexity of *u*_*L*_ reflect the different behaviors, either averting or seeking risk, when the object is framed in terms of gain or loss, respectively, with respect to some reference point (Fig. 1(a)). It also reflects the bias in sensitivity due to framing, to lose some amount rather than to gain the same amount, as a result of the inclusion of the loss aversion index λ.

The weighing of the likelihood of an event happens in a two-stage process^51^. First the decision maker estimates the probability of the event (*p*), and second he/she assigns a weight to it (*ω*(*p*)). The weight function should reflect the compression effect for the objective or true probabilities. That compression results in an overestimation of low probabilities and underestimation of high probabilities. The weight function *ω* has a number of other properties: sub-certainty or *ω*(*p*) + *ω*(1 − *p*) < 1, and the pseudo-certainty feature of human cognition: people value a decrease in probability from 0.1 to 0 more than they value a decrease from 0.2 to 0.1, for example. An example of the weight function *ω* of a probability *p* is depicted in Fig. 1(b), and the function is given by


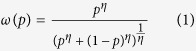


where 0 < *η* < 1 2,52. The case 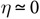 corresponds to complete denial of the event unless it is certain. We will call the parameters *α*, *λ*, and *η* the cognitive parameters.

### Behavior-disease model

We build a behavior-disease model that couples disease transmission dynamics to vaccinating behavior dynamics (see e.g.^25^,^35^), in which susceptible babies are born at a rate of *μ* (1 − *xe*) where *μ* is the birth/death rate, *x* is the proportion of vaccinators (and rate of vaccination) and *e* is the vaccine efficacy. The model is intended to apply to paediatric infectious diseases such as measles, pertussis, and chicken pox. The model is given by


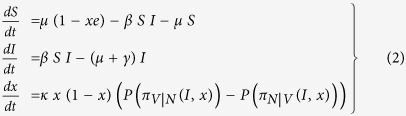


where *S* and *I* are the proportions of susceptible and infected individuals in the population, *β* is the disease transmission rate, and *γ* is the recovery rate. (The differential equation for the proportion of recovered individuals *R* is redundant and so does not appear.) A parent is either a vaccinator *V* or non-vaccinator *N*. In the behavior equation of the model, parents sample one another at a rate of *κ* and compare their payoff to the sampled parent’s payoff; then they pick up the other parent’s strategy with a probability given by a function in the difference of the payoffs. A non-vaccinator parent perceives a difference between vaccination and non-vaccination strategies of *π*_*V*|*N*_ = *π*_*N*_(*V*) − *π*_*N*_(*N*) and a vaccinator parent perceives a difference between non-vaccination and vaccination strategies of *π*_*N*|*V*_ = *π*_*V*_(*N*) − *π*_*V*_(*V*) (see below for their definitions). Both are dependent on the disease prevalence *I* and the rate of vaccination *x*.

The probability function *P* is defined by 
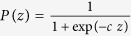
 for some constant *c* > 0 (see^32^) so that the larger the value of *z* is, the closer the value of the function *P*(*z*) is to one. *P*(*π*_*V*|*N*_) is the probability that a parent switches from non-vaccinator to vaccinator strategy and *P*(*π*_*N*|*V*_) is the probability that a parent switches from vaccinator to non-vaccinator strategy.

Within each of the two groups *V* and *N*, it is expected that due to the omission bias^53^ (harms of commission exceeds the harms of omission) parents might show different loss aversion parameters *λ* when considering risk of vaccination versus risk of non-vaccination. Let the subscripts *c* and *o* be used to emphasize the commission and omission of actions. While the relationship between values of the loss aversion parameters *λ*_*c*_ and *λ*_*o*_ is unclear in the case of vaccination decision-making, it is valid to postulate that the loss aversion parameter of commission is greater than that of omission. That is, if two prospects of omission and commission are of the same outcomes and probabilities then the utility of the prospect due to commission would be greater than of the prospect due to omission if and only if *λ*_*c*_ > *λ*_*o*_. While the values of the loss parameters will not be restricted to that inequality, as it seems that the omission bias in the underlying problem is complicated by the values of the prospect.

We assume that all babies are born healthy and their parents’ goal is to maintain the healthy status of their children. When an individual encounters some information about vaccination or disease, he/she reflects upon it from his/her own perspective (or current strategy). Then, the utilities will be given by


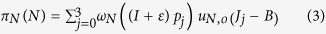



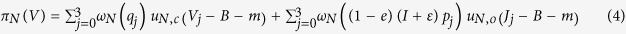



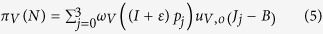



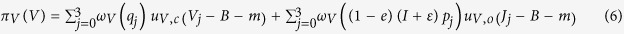


Here, *B* = 1 is the utility of the healthy status (the reference point); *J*_0_, *J*_1_, *J*_2_, *J*_3_ are the utilities of mild, moderate, morbid, and death due to disease that occur with probabilities *p*_0_, *p*_1_, *p*_2_, *p*_3_ to an infected child such that 1 = *B* ≥ *J*_0_ > *J*_1_ > *J*_2_ > *J*_3_ ≥ 0. The quantities *V*_0_, *V*_1_, *V*_2_, *V*_3_ are the utilities of no-side-effect, mild, morbid, and death due to adverse reaction to vaccination that occur with probabilities *q*_0_, *q*_1_, *q*_2_, *q*_3_ to a vaccinated child such that 1 = *B* ≥ *V*_0_ > *V*_1_ > *V*_2_ > *V*_3_ ≥ 0. The quantity *m* is the utility (normalized and given in utils) of cost and effort to access vaccination. (We assume that all of the utilities are in units of ‘utils’.) The parental perception of the probability of getting infected is proportional to the current prevalence *I* offset by a small probability 0 < *ε* << 1 making a total of *I* + *ε* to account for that at zero prevalence there might be a very faint fear of infection due to immigration. (The quantity *ε* is also mathematically important in the analysis of the model.) The quantity (1 − *e*)(*I* + *ε*)*p*_*j*_ is the probability that a vaccinated child gets a disease reaction of type *J*_*j*_ after getting infected by internal or external sources of infection as the vaccine fails to work. The vaccine efficacy will always affect the disease incidence. It will, however, have effect on vaccination behavior in the bounded rational case only (see results).

Social norms and peer group pressure are important factors in immunization uptake^33^,^54^. Therefore we postulate the following: each group adopting a strategy Θ imposes a social group pressure given by *δ*_Θ_ on the population. Thus an individual adopting strategy Θ experiences an average social pressure given by *p*_Θ_ *δ*_Θ_, where *p*_Θ_ is the proportion of strategy Θ′s adopters/supporters. The group pressures *δ*_*V*_ and *δ*_*N*_ are normalized to be between 0 and 1 and are given in ‘utils’. Hence,


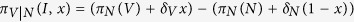



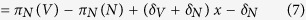



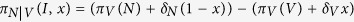



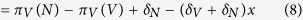


where *π*_*N*_(*V*), *π*_*N*_(*N*), *π*_*V*_(*N*), and *π*_*V*_(*V*) are given by equations (3), (4), (5), (6). This means that the utility of the vaccination prospect *π*_Θ_(*V*) is augmented by the amount of the vaccinators’ group pressure *δ*_V_ *x* and the utility of the non-vaccination prospect *π*_Θ_(*N*) is augmented by the non-vaccinators’ group pressure *δ*_*N*_ (1 − *x*).

## Results

The “rational decision model” is recovered as a special case of the full cognitive behavior-disease model, in which the cognitive parameters, *α*_Θ_, *η*_Θ_, and 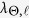 for Θ = *V*,*N* and 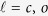 are set equal to one (see Table 1 in Appendix I). However, we point out that even the “rational decision model” is not a pure rational decision model since it allows for processes of social learning and social norms. Therefore our use of the term is relative. The rational decision model is identical to the model in^33^ in case of perfect vaccine efficacy and equal group pressure. When the parameters *α*_Θ_, *η*_Θ_, and 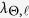 are not equal to one, we refer to the model as a “bounded-rational decision model”.

In our analysis, we explore what happens as the parameters *α*_Θ_, *η*_Θ_, and 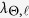 move away from the rational decision model to the bounded rational decision model. This results in a plethora of dynamical behaviors of vaccine acceptance, giving rise to new model equilibria relative to the rational case^33^. We explore how dynamics depend on vaccine efficacy, vaccine cost, and social norms.

The model (equation (2)) has six fixed points. Three fixed points are disease-free equilibria: 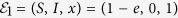 (pure vaccinator, disease-free), 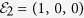 (non-vaccinator, disease-free), and 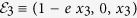 (partial vaccinator, disease-free; *x*_3_ is a function of various model parameters, and its full expression appears in Appendix II).

The remaining three fixed points are disease-endemic equilibria that depend on the quantity 
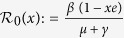
. 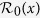 is an effective reproduction number in the presence of a partially immune population. Hence, the basic reproduction number *R*_0_ is given by 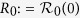. The three endemic equilibria are: 
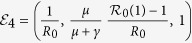
 (pure vaccinator, disease-endemic), 
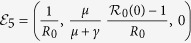
 (no vaccinator, disease-endemic), and 
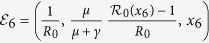
 (partial vaccinator, disease-endemic; see Appendix II for a definition of *x*_6_).

Details about the existence of the equilibrium points and their stability conditions appear in Appendix II, but we summarize the findings in this and the following paragraphs. The dependence of the equilibria on model parameters is generally intuitive. The pure vaccinator, disease-free equilibrium (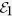) is stable if the disease is not highly contagious and the vaccine is not very scary compared to the strength of the vaccinator group pressure (the average vaccine risk with immigration (see Appendix II) is less than the vaccinator group pressure). In contrast, the non-vaccinator, disease-free equilibrium (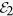) is stable when the disease cannot sustain its spread and non-vaccinator pressure is considerable. The partial vaccinator, disease-free equilibrium (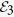) is not stable whenever it exists, because in the absence of disease, social norms will always move vaccine coverage up or down from an intermediate vaccine coverage, hence the steady state is unstable.

The pure vaccinator, disease-endemic equilibrium (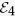) is stable if and only if vaccinator pressure is sufficiently large and the vaccine efficacy is not too large (otherwise, the disease would be eliminated). That is, under the appropriate vaccinator pressure, the vaccine can be used to mitigate or completely eliminate a disease depending on its efficacy. The non-vaccinator, disease-endemic equilibrium (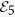) is stable if and only if the non-vaccinator pressure is sufficiently large. Finally, the partial vaccinator, disease-endemic equilibrium (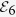) is stable under some highly specific technical conditions (see Appendix II).

The vaccine should be supported by a vaccinator social pressure larger than the average vaccine risk with immigration (see Appendix II). This was also found by Oraby *et al.*^33^, but for the rational decision model and a completely efficacious vaccine. However, in the current model, the average vaccine risk depends on the cognition of the parents and in many cases, the (perceived) vaccine risk is increasing as the cognitive parameters move away from the rational decision model (Appendix IV, Figures A1-A6). Additionally, in the current model, incorporating vaccine efficacy as a parameter resulted in the emergence of a new equilibrium point (pure vaccinator, disease-endemic)^33^. This new equilibrium can be maintained if the average vaccine risk at the disease-endemic prevalence is less than the social pressure of vaccinators. Again, the vaccine risk depends on the cognitive parameters and in many cases it increases as the cognitive parameters move away from the rational decision model (see Appendix IV Figures A1-A6). If the vaccine is sufficiently efficacious, full vaccine coverage can eliminate the infection.

### Simulation results

Numerical simulations presented via parameter planes that show the various dynamical regimes of the model provide a clearer picture of the impact of introducing bounded cognitive processes. For baseline parameter values of the numerical simulation, we use a basic reproduction number *R*_0_ = 17, a birth/death rate of *μ* = 1/50 year^−1^, and a recovery rate of γ = 365/22 year^−1^ from published estimates of pertussis^25^. According to the Centers for Disease Control and Prevention, approximately 1 in 8 of children infected by pertussis suffers from pneumonia, 1 in 20 suffers from encephalitis, and 1 in 1,500 dies^55^. We assume that only the severest disease outcomes was reported (so the events were mutually exclusive); then we take *p*_1_ = 1/8, *p*_2_ = 1/20, *p*_3_ = 1/1500 and *p*_0_ = 1 − (*p*_1_ + *p*_2_ + *p*_3_). The diphtheria, tetanus, and pertussis acellular vaccine (DTaP) can cause side effects from mild reactions to acute encephalopathy. No death has been proven from DTaP vaccination so far. It can, however, cause continuous crying followed by full recovery for 1 in 1,000 vaccinated children, convulsions or shock followed by full recovery for 1 in 14,000, and acute encephalopathy for 0-10.5 in 1,000,000 55. We assume that *q*_1_ = 1/1000, *q*_2_ = 1/14000, *q*_3_ = 10.5/1000000 (the worst case) and *q*_0_ = 1 − (*q*_1_ + *q*_2_ + *q*_3_). Since there is no documented objective measure of the quality of life of repercussions for the disease and vaccination, we assume that *V*_0_ = .99, *V*_1_ = .7, *V*_2_ = .3, and *V*_3_ = .1; whereas *J*_0_ = .9, *J*_1_ = .5, *J*_2_ = .1, and *J*_3_ = 0. We choose a negligible *ε* of value 10^−20^. These parameter values are summarized in Appendix I, Table 1. The cognitive parameters, *α*_Θ_, *η*_Θ_, and 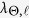 for Θ = *V*,*N* and 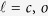, see Table 1 in Appendix I, are assigned different sets of values to showcase the different dynamical behaviors of the vaccination rates and incidence.

**Figure 2:**
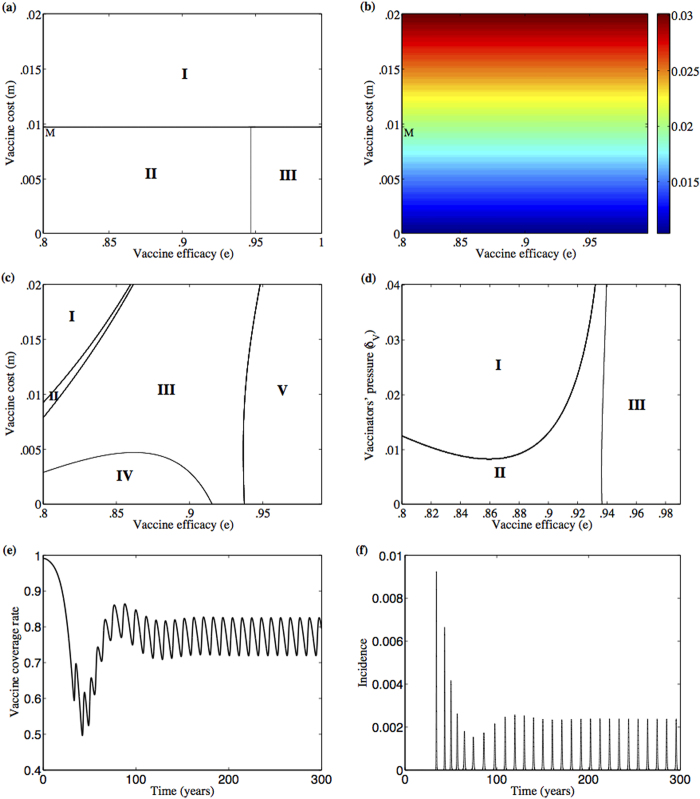
Various dynamical behaviors of vaccine uptake emerge at different values of vaccine cost and efficacy in the rational decision model. (**a**,**b**) **and the bounded-rationality model** (**c**,**d**). *e*-*m* plane at *δ*_*V*_ = 0.02 of the rational decision model (**a**) and the bounded-rationality case (**c**,**d**) at cognitive parameters *α*_*V*_ = 0.8271, *α*_*N*_ = 0.9480, *λ*_*V*,*o*_ = 9.4515, *λ*_*V*,*c*_ = 1.7600, *λ*_*N*,*o*_ = 1.9011, *λ*_*N*,*c*_ = 1.7438, *η*_*V*_ = 0.2827, *η*_*N*_ = 0.5114. In (**a**) there are two bistability regions where the equilibrium points 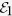 and 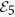 are stable in region III, and the equilibrium points 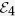 and 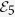 are stable in region II. Besides, equilibrium point 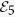 is stable in region I and in an extremely narrow stripe between the two bistability regions II and III (bounded by *e* = 0.941159 and *e* = 0.941176). *M* = .00975 is the cutoff vaccine cost in case *δ*_*V*_ = 0.02 in (**a**) (see also panel (**b**)). (**b**) A contour plot for values of 
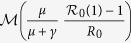
 calculated at each pair of (*e*,*m*) in the rational decision model. The full vaccination equilibrium point 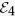 is stable in the regions where *δ*_*V*_ is larger than the value of 
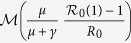
 and equation (8) in Appendix II is valid, that is *e* < 0.941159. With deviation from the rational decision model, a different *e*—*m* plane emerges in (**c**). There, a bistability region (II) of the equilibrium points 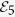 and 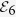 transpires as well as other regions of stability for 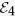 in IV, 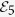 in I, and 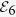 in III. Limit cycles appear in region V. (**d**) *e*—*δ*_*V*_ plane of stability, at *m* = 0, in which the equilibrium point 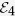 is stable in region I and 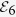 in region II while limit cycles appear in region III. In both (**c**) and (**d**), the line between the regions where 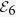 is stable and the limit cycles is a supercritical Hopf bifurcation line whereas the rest of the lines are stability changing bifurcation lines. Limit cycles of vaccine coverage rate (**e**) and incidence (**f**) at *δ*_*V*_ = 0.02, *m* = 0, and *e* = 0.99. The rest of the parameters are *κ* = 1.69,*c* = 1.46,*δ*_*N*_ = 0.02 in all of the subpanels.

Under the rational decision model, at baseline parameter values, there are three parameter regimes in the *m* − *e* parameter value (Fig. 2(a)), meaning that three different types of dynamics can emerge depending on parameter values. A sufficiently costly vaccine (large *m*, as might apply at times of war, for example, when the vaccine cost and accessibility are large) will always result in zero vaccine coverage. However, if the vaccine is less expensive, then two regions of bistability (regions II and III) are encountered, where the outcome can either be no vaccination or full vaccination in each one of them, depending on the initial conditions (Fig. 2(a)). The bistability emerges because social norms can drive vaccine coverage up or down, depending on whether vaccinators or non vaccinators are initially more numerous. Moreover, in the bistability region II, if vaccine efficacy is sufficiently small, then the disease can become endemic despite full vaccination, whereas if vaccine efficacy is sufficiently large (region III), then the disease can be eradicated in the presence of full vaccination (Fig. 2(a)). The cut-off point (M) in vaccine cost *m* that separates the region of no vaccination and the region of bistability (full or no vaccination) depends on the amount of vaccinator pressure *δ*_*V*_ (Fig. 2(b)). At sufficiently large vaccine efficacy and sufficiently large initial acceptance of vaccine, the effect of larger costs can be offset by sufficiently large group pressure, resulting in disease eradication (Fig. 2(b)).

**Figure 3:**
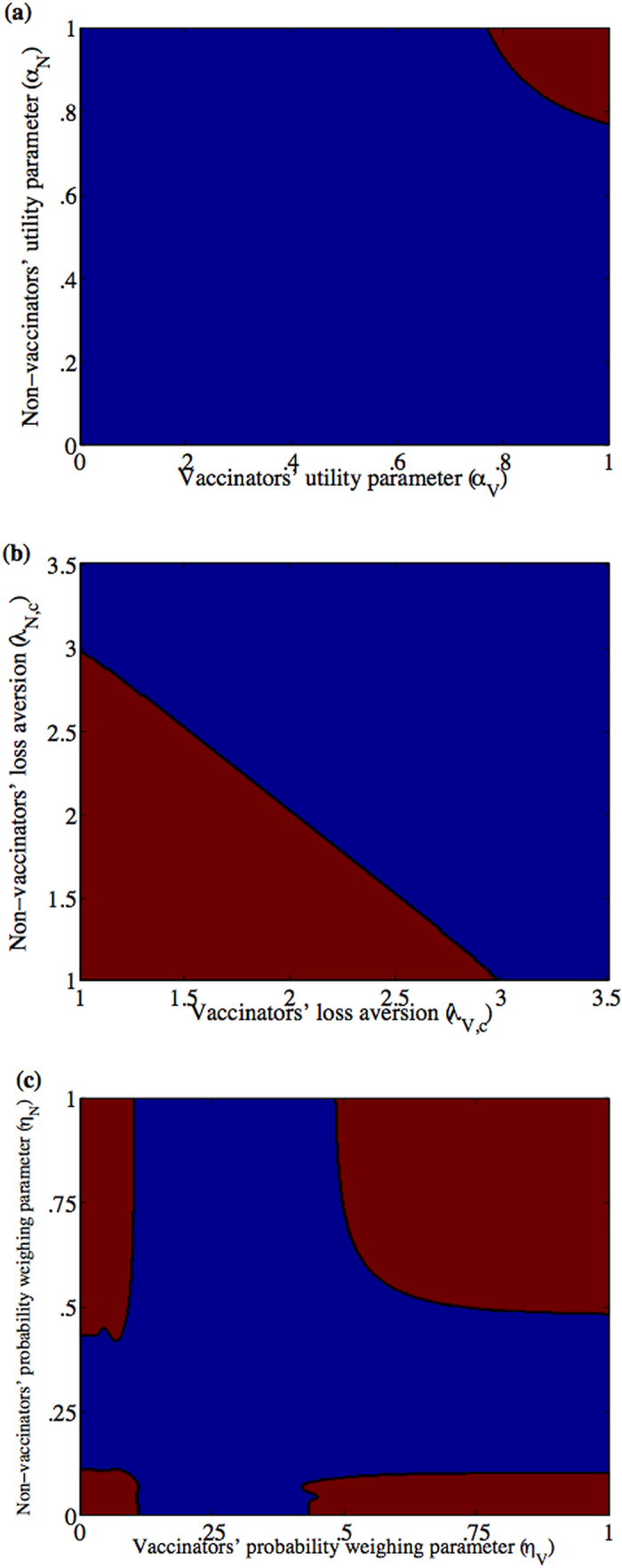
Deviations from the rational decision model influence the vaccine uptake levels. (**a**) *α*_*V*_—*α*_*N*_ plane, (**b**) *λ*_*V*,*c*_—*λ*_*N*,*c*_ plane with *λ*_*V*,*o*_ = *λ*_*V*,*c*_ and *λ*_*N*,*o*_ = *λ*_*N*,*c*_, and (**c**) *η*_*V*_—*η*_*N*_ plane at *m* = 0 and *δ*_*V*_ = 0.02. The equilibrium points 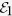 (pure vaccinator, disease-free) and 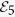 (no vaccinator, disease-endemic) are stable in the red region which includes the point (1,1)—corresponding to the rational decision model, given the values of the rest of the cognitive parameters equal to one–whereas in the blue region the only stable point is 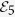. The rest of the parameters are *κ* = 1.69, *c* = 1.46, *e* = .95.

**Figure 4:**
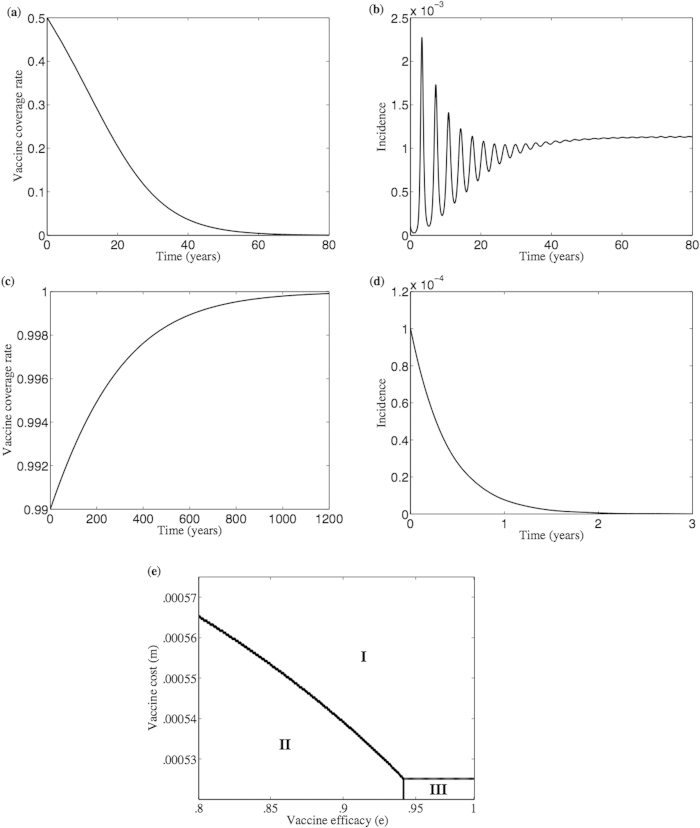
Vaccine acceptance rate and disease incidence depend on the initial vaccine coverage and its efficacy. Simulation of both vaccine coverage rate (**a**,**c**) and disease incidence (**b**,**d**) when initial vaccine coverage is 50% (**a**) and 99% (**c**) at vaccine efficacy *e* = 0.95 and cost *m* = 0 which lie in the extension of region III in subpanel (**e**) of the *e*—*m* plane of a bounded rational model. In (**e**) there are two bistability regions where the equilibrium points 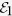 and 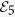 are stable in region III, and the equilibrium points 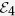 and 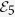 are stable in region II. Besides, equilibrium point 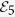 is stable in region I and in an extremely narrow stripe between the two bistability regions II and III. Compare subpanel (**e**) to Fig. 2(a). The lines in (**e**) are stability changing bifurcation lines. The rest of the parameters are *κ* = 1.69, *c* = 1.46, *δ*_*V*_ = 0.0725, *δ*_*N*_ = 0.02, *α*_*V*_ = 0.9715, *α*_*N*_ = 0.9379, *λ*_*V*,*o*_ = 6.9507, *λ*_*V*,*c*_ = 9.4554, *λ*_*N*,*o*_ = 5.8909, *λ*_*N*,*c*_ = 1.9230, *η*_*V*_ = 0.9882, *η*_*N*_ = 0.9362.

When the cognitive parameters *α*_Θ_, *η*_Θ_, and 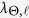 are changed such that the system moves from the rational decision model to the bounded rationality model (as depicted in Fig. 3; see explanation below), the model predictions change dramatically. This is visible upon a casual inspection of the parameter regimes for the bounded rational model case (Fig. 2(c)) versus the rational decision model case for a range of values of the vaccine cost *m* and efficacy *e* (Fig. 2(a)); we observe that the number of different possible dynamical behaviours has increased, and the boundaries between the different regions have become nonlinear (see also Figs. 2(d) and 4(e) for the bounded rational model). Hence, a wider range of population behaviours are possible, when individuals are subjected to bounded cognitive processes. The pure vaccinator, disease-free equilibrium is replaced by a region of limit cycles (region V in Fig. 2(c)), where vaccine coverage and the proportion of vaccinators in the population oscillate over time (Fig. 2(c) versus 2 (a), see also Fig. 2(d,e)). A stable region of partial vaccination (region III in Fig. 2(c)), which shares a bistability region (region II) with null vaccination, emerges as well. The region of full vaccination and endemic disease (region IV) shrinks. For another choice of cognitive parameters, the shape of the pure vaccinator, disease endemic stability region (region II in Fig. 4(e)) changes shape at a different vaccine cost. The vaccine may be fully accepted within a range of vaccine efficacy similar to that of DTaP vaccine (from 80% to 90%^56^) but at low cost of vaccination (see Fig. 2 (c) and 4(e)). The disease, however, cannot be eradicated because of insufficient vaccine efficacy. Introducing bounded cognitive processes in particular has, therefore, removed the possibility of disease eradication under voluntary vaccination, at least for the baseline parameter values (Appendix I Table 1).

Next, the relationship between the rational model and the bounded rational model is further explored, by determining what happens when the cognitive parameters *α*_Θ_, *η*_Θ_, and 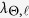 are changed gradually, starting from values corresponding to the rational model and moving to values corresponding to the bounded rational model. We plotted a series of parameter planes where all the cognitive parameters are fixed at unity, except for those which are being varied along the axes of the parameter planes across a range including the unitary values corresponding to the rational decision model (Fig. 3). In particular, stability regions of the *α*_*V*_–*α*_*N*_, *λ*_*V*,*c*_–*λ*_*N*,*c*_, and *η*_*V*_–*η*_*N*_ planes are explored at vaccine cost *m* = 0 and vaccine efficacy *e* = .95 (Fig. 3). In all of the figures, the non-vaccinator, disease-endemic equilibrium (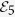) is always stable, whatever was the group pressure, in contrast to the rational decision model in^33^ where it is stable in a limited region. The pure vaccinator, disease free equilibrium (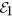) appears as another stable equilibrium in a region containing the rational decision model, which is represented by the pair (1,1) in the parameter plane. That region depends on the amount of group pressure (Appendix IV for Figures A1, A2, and A3). In other words, given a fixed group pressure, shifting away enough from the pair (1,1) (the rational decision model) and into the bounded rational model, makes it impossible to achieve any vaccine acceptance. The size of that region is also dependent on the vaccine cost (see also Appendix IV for Figures A1, A2, and A3 for vaccine cost *m* = 0,.01, and .02). Similar conclusions follow when efficacy *e* = .9 but then the pure vaccinator, disease endemic equilibrium (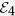) appears in lieu of 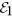 and so the disease cannot be eradicated at all (see Appendix IV for Figures A4, A5, and A6 when *m* = 0,.01, and .02). In general, introducing bounded rationality into the model makes it harder to eradicate the infection (compare the blue regions of endemic disease, which occur away from the rational (1,1) case, to the red regions containing (1,1) in Figures A1, A2, A4, A5), although there are exceptions (Figures A3 and A6). In the latter two figures the closer the value of *η*_*V*_ and/or *η*_*N*_ to zero, the more the denial of the adverse event, unless it is absolutely certain (has 100% likelihood to occur). That would make the value of the average perceived risk of vaccine (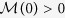, see Appendix II for definition) very small and so parents will accept the vaccine if there is a large initial proportion of vaccinators.

As noted above, under the bounded rationality model, in contrast to the rational decision model, increasing vaccine efficacy may lead to oscillations instead of eradication (see the *e*–*m* plane in Fig. 2(c) versus 2(a) and the *e*–*δ*_*V*_ plane in Fig. 2(d)). Moreover, within a range of vaccine efficacy similar to that of the DTaP, increasing vaccinator pressure can help in reaching full vaccine coverage, but it only reduces disease prevalence by a factor 
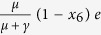
 (which, here, is strictly less than 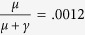) rather than leading to full elimination. On the other hand, at a different set of parameter values in which the vaccinator group pressure instead exceeds the non-vaccinator group pressure, it is sometimes impossible to achieve high level of vaccine acceptance if the size of vaccinator group was not initially large enough (see for example Fig. 4(a,b) when the initial vaccination rate is 50%). However, for a larger initial vaccinator group sizes, the disease may be eliminated for the same cognitive parameters (see for example Fig. 4(c,d) when the initial vaccination rate is 99%).

## Discussion

Research in cognitive psychology shows that humans make conflicting decisions when the same problem is re-framed, not to mention the effect of other biases and fallacies on those decisions. Here, we introduce a model of vaccine decision-making that includes bounded rationality, social norms, vaccine efficacy and vaccine cost. In addition to social group pressure and the disease- specific parameters, the model includes three cognitive parameters for each group of vaccinators and non-vaccinators. The model generalizes the model in^33^ by adding cognitive parameters and allowing vaccine efficacy to be less than perfect.

We find that richer dynamical behavior emerges from our model, in contrast to corresponding models that are closer to a pure “rational actor” model lacking bounded rationality and in which vaccine efficacy is 100% (33). Allowing vaccine efficacy to be less than 100% causes the appearance of a new equilibrium in which endemic disease with full vaccine acceptance becomes stable. It also transforms the possible vaccine coverage level to a range from anywhere from zero to full coverage. Moreover, generally speaking, introducing bounded rationality makes it more difficult to eliminate the infection under a non-mandatory vaccination policy. This echoes many previous findings that introducing individual decision-making mechanisms could make it more difficult to eliminate an infection^35^,^40^,^41^,^43^,^44^,^48^. However, injunctive social norms can correct for the deleterious effects of cognitive processes on vaccine coverage. Combinations of cognitive parameters and group pressure can largely determine the vaccine uptake. Non-vaccinator group pressure, however, has almost no effect on the vaccination dynamics at some selected disease parameters. In addition, vaccine cost and efficacy have a significant effect on vaccine acceptance rates.

While full vaccine refusal is always a stable equilibrium in a rational decision model^33^,^35^, a bounded rationality model can change the parameter regime where it is stable. Bounded rationality also gives rise to stable limit cycles and partial vaccination levels. Parameter values close to those of a rational decision model in addition to high group pressure can lead to full vaccine acceptance if the initial size of vaccinator group is large enough. Nonetheless, a very highly efficacious vaccine might be needed to eradicate the disease. Vaccine cost and accessibility can lead to low rates of vaccine acceptance if vaccinator pressure is not large enough.

The model introduced here has some challenges and limitations. First, the model has many cognitive parameters, which hinders their estimation with the currently available data on vaccinating behaviour for common paediatric infectious diseases. This also affects our understanding of the relative importance of social versus cognitive components in parental vaccination decisions. Our simulations, however, suggest that injunctive social norms can counteract the effect of (mis)perception of risks, high vaccination cost, and low vaccine efficacy. The model did not also include age structure to differentiate between contact rates between children and between adults^57^. Age structure could be important for certain research questions. Also, the model does not account for social network structure, and/or stochastic, small population processes that may emerge close to the eradication threshold due to unanticipated interactions between social structure, decision-making, and stochastic disease dynamics. Finally, previous experience with vaccination or infection influences individual decision-making but we have not included such effects here^58^. These and other effects could be best explored in future work using a individual-based network simulation model.

The role of omission bias in vaccine acceptance and its representation in mathematical models merits further investigation. Here, we tried to address omission bias by using different loss aversion parameters for omission and commission. However, this is a simplification of omission bias. It would be also interesting to explore whether declining vaccination for the non-vaccinator group is considered an omission or, from their point of view, it is a “commission” of the right thing. This kind of argument follows from some early results finding that omission bias is not a driver of vaccine exemption^59^.

There has always been a need to understand how social and cognitive components affect parental decisions towards vaccination. Our results show how combinations of both sets of parameters—along with the vaccine efficacy, cost and the rates of vaccine acceptance at the beginning of the vaccination campaign—can determine the fate of vaccine acceptance. Empirical validation of such models developed for specific paediatric infectious diseases could help health authorities identify vaccine programs that might be more prone to vaccine refusal in the future, and thus help authorities determine how to prioritize risk messaging in light of knowledge of how individuals can sometimes mis-perceive risks.

## Additional Information

**How to cite this article**: Oraby, T. and Bauch, C. T. Bounded rationality alters the dynamics of paediatric immunization acceptance. *Sci. Rep.* **5**, 10724; doi: 10.1038/srep10724 (2015).

## Supplementary Material

Supplementary Information
