# Supplementary material for: Bounded rationality alters the dynamics of paediatric immunization acceptance
Source: Sci Rep. 2015 Jun 2;5:10724. doi: 10.1038/srep10724 (PMC4451793; doi:10.1038/srep10724)
Supplement: Supplementary Information [file srep10724-s1.pdf]

Bounded Rationality Alters the Dynamics of Paediatric Immunization Acceptance

Tamer Oraby[1,*], Chris T. Bauch[2]

1 Department of Mathematics, University of Texas – Pan American, Edinburg, Texas, USA,

2 Department of Applied Mathematics, University of Waterloo, Waterloo, Ontario, Canada

* E-mail: orabytf@utpa.edu

Appendix I: Table of parameters

Table 1. Model parameters and symbols with their definition.

Family	Parameters/ Symbols	Definition	Baseline values or ranges	Ref.
Vaccine	e	Vaccine efficacy	$.8-1$	Assumed
	m	Vaccine cost and accessibility	$0-.02$ utils	Assumed
	V_i	Vaccine adverse event i $(i=0,\ldots,3)$	$(.99,.7,.3,.1)$ utils	Assumed
	q_i	Probability of V_i's occurrence	$q_0=1/1000, q_1=1/14000,$<br>$q_2=10.5/1000000$<br>$q_3=1-(q_0+q_1+q_2)$	[1]
Disease	β	Transmission rate	282.385 year^{-1}	[2]
	γ	Recovery rate	365/22 year^{-1}	[2]
	J_i	Disease adverse event i $(i=0,\ldots,3)$	$(.9,.5,.1,0)$ utils	Assumed
	p_i	Probability of J_i's occurrence	$p_0=1/8, p_1=1/20,$<br>$p_2=1/1500,$<br>$p_3=1-(p_0+p_1+p_2)$	[1]
Demographic	μ	Birth/death rate	1/50 year^{-1}	[2]
Behavior	κ	Imitation rate	1.69 year^{-1}	Assumed
	c	Slope of logit(P)	1.46	Assumed
Social	δ_Θ	Social support of group Θ $(\Theta=V,N)$	$0-.1$ utils	Assumed
Cognitive	α_Θ	Utility function exponent of group Θ $(\Theta=V,N)$	$0-1$	[3,4]
	$\lambda_{\Theta,\ell}$	Loss aversion of group Θ with $\ell=c$ (commission), o (omission)	$1-\infty$	[3,4]
	η_Θ	Parameter of weight function of group Θ	$0-1$	[4,5]

Appendix II: Model equilibria and stability analysis

The model

The model is given by

$$\left.\begin{aligned}\frac{dS}{dt} &= \mu\,(1-xe) - \beta\,S\,I - \mu\,S\\ \frac{dI}{dt} &= \beta\,S\,I - (\mu+\gamma)\,I\\ \frac{dx}{dt} &= \kappa\,x\,(1-x)\,\big(P(\pi_{V|N}(I,x)) - P(\pi_{N|V}(I,x))\big)\end{aligned}\right\} \tag{1}$$

where S and I are the proportions of susceptible and infected individuals in the population, μ is the birth/death rate, γ is the recovery rate, e is the vaccine efficacy, β is the disease transmission rate, x is the proportion of vaccinators (and rate of vaccination) and κ is the imitation rate.

The probability function P is defined by $P(z) = \frac{1}{1+\exp(-c\,z)}$ for some constant $c > 0$. The utilities are given by

$$\pi_N(N) = \sum_{j=0}^{3} \omega_N((I+\varepsilon)\,p_j)\,u_{N,o}(J_j - B) \tag{2}$$

$$\pi_N(V) = \sum_{j=0}^{3} \omega_N(q_j)\,u_{N,c}(V_j - B - m) + \sum_{j=0}^{3} \omega_N((1-e)\,(I+\varepsilon)\,p_j)\,u_{N,o}(J_j - B - m) \tag{3}$$

$$\pi_V(N) = \sum_{j=0}^{3} \omega_V((I+\varepsilon)\,p_j)\,u_{V,o}(J_j - B) \tag{4}$$

$$\pi_V(V) = \sum_{j=0}^{3} \omega_V(q_j)\,u_{V,c}(V_j - B - m) + \sum_{j=0}^{3} \omega_V((1-e)\,(I+\varepsilon)\,p_j)\,u_{V,o}(J_j - B - m) \tag{5}$$

$B = 1$ is the utility of the healthy status (the reference point); J_0, J_1, J_2, J_3 are the utilities of mild, moderate, morbid, and death due to disease that occur with probabilities p_0, p_1, p_2, p_3 to an infected child such that $1 = B \geq J_0 > J_1 > J_2 > J_3 \geq 0$. The quantities V_0, V_1, V_2, V_3 are the utilities of no-side-effect, mild, morbid, and death due to adverse reaction to vaccination that occur with probabilities q_0, q_1, q_2, q_3 to a vaccinated child such that $1 = B \geq V_0 > V_1 > V_2 > V_3 \geq 0$. The quantity m is the utility (normalized and given in utils) of cost and effort to access vaccination.The parental perception of the probability of getting infected is proportional to the current prevalence I offset by a small probability $0 < \varepsilon << 1$ making a total of $I + \varepsilon$ to account for that at zero prevalence there might be a very faint fear

of infection due to immigration.

Model equilibria

Moving away from the rational decision model with social norms by adding a cognitive dimension to the model results in a plethora of dynamical behaviors of vaccine acceptance, by giving rise to new model equilibria relative to [6]. In addition to vaccine acceptance, incorporating vaccine efficacy and vaccine cost into the model has an effect on the disease dynamics. Social norms can rectify all deviations from the rational decision model of a partially-efficacious costly vaccine.

Let

$$\begin{aligned}\mathcal{M}(y) \quad := \quad & \frac{1}{2}\Bigg[-\sum_{j=0}^{3}\omega_V(q_j)\,u_{V,c}(V_j-B-m)-\sum_{j=0}^{3}\omega_N(q_j)\,u_{N,c}(V_j-B-m) \\ & +\sum_{j=0}^{3}\omega_V((y+\varepsilon)\,p_j)\,u_{V,o}(J_j-B)+\sum_{j=0}^{3}\omega_N((y+\varepsilon)\,p_j)\,u_{N,o}(J_j-B) \\ & -\sum_{j=0}^{3}\omega_V((1-e)\,(y+\varepsilon)\,p_j)\,u_{V,o}(J_j-B-m)-\sum_{j=0}^{3}\omega_N((1-e)\,(y+\varepsilon)\,p_j)\,u_{N,o}(J_j-B-m)\Bigg]\end{aligned}$$

be the simple average of both groups' gain/loss of adopting the strategy of not-to-vaccinate when the disease prevalence is y. Here, when $\varepsilon = 0$ (which is almost the case), $\mathcal{M}(0)$ is the average vaccine risk perceived by vaccinators and non-vaccinators. Thus, we will call $\mathcal{M}(0)$ is the average vaccine risk with immigration.

The model equation (1) has six fixed points. Three fixed points are disease-free equilibria: $\mathcal{E}_1 = (S, I, x) = (1-e, 0, 1)$, $\mathcal{E}_2 = (1, 0, 0)$, and $\mathcal{E}_3 \equiv (1-e\,x_3, 0, x_3)$, where x_3 solves the equation $P(\pi_{V|N}(0, x_3)) = P(\pi_{N|V}(0, x_3))$ or simply $\pi_{V|N}(0, x_3) = \pi_{N|V}(0, x_3)$ according to the definition of P. Hence $x_3 = \frac{1}{\delta_N+\delta_V}(\delta_N + \mathcal{M}(0))$ and so the equilibrium point $\mathcal{E}_3$ exists if and only if $\delta_N > -\mathcal{M}(0)$ when $\mathcal{M}(0) < 0$ or $\delta_V > \mathcal{M}(0)$ when $\mathcal{M}(0) > 0$ (or simply, $\delta_V > \mathcal{M}(0) > -\delta_N$).

Let the effective reproduction number $\mathcal{R}_0(x) := \frac{\beta\,(1-xe)}{\mu+\gamma}$ and let the basic reproduction number $R_0 := \mathcal{R}_0(0)$. The remaining three fixed points are disease-endemic equilibria: $\mathcal{E}_4 = (\frac{1}{R_0}, \frac{\mu}{\mu+\gamma}\,\frac{\mathcal{R}_0(1)-1}{R_0}, 1)$, $\mathcal{E}_5 = (\frac{1}{R_0}, \frac{\mu}{\mu+\gamma}\,\frac{\mathcal{R}_0(0)-1}{R_0}, 0)$, and $\mathcal{E}_6 = (\frac{1}{R_0}, \frac{\mu}{\mu+\gamma}\,\frac{\mathcal{R}_0(x_6)-1}{R_0}, x_6)$, where x_6 solves the equation

$$P\left(\pi_{V|N}\left(\frac{\mu}{\mu+\gamma}\,\frac{\mathcal{R}_0(x_6)-1}{R_0}, x_6\right)\right) = P\left(\pi_{N|V}\left(\frac{\mu}{\mu+\gamma}\,\frac{\mathcal{R}_0(x_6)-1}{R_0}, x_6\right)\right)$$

which according to the definition of P is just the solution of

$$\pi_{V|N}\left(\frac{\mu}{\mu+\gamma}\,\frac{\mathcal{R}_0(x_6)-1}{R_0},x_6\right)=\pi_{N|V}\left(\frac{\mu}{\mu+\gamma}\,\frac{\mathcal{R}_0(x_6)-1}{R_0},x_6\right)$$

or the intersection of the line $(\delta_V+\delta_N)\,x-\delta_N$ and the function g defined by $g(x):=\mathcal{M}(\frac{\mu}{\mu+\gamma}\,\frac{\mathcal{R}_0(x)-1}{R_0})$.

Equilibrium point $\mathcal{E}_4$ exists if and only if $\mathcal{R}_0(1)>1$ (or $e<1-\dfrac{1}{R_0}$), whereas $\mathcal{E}_5$ exists if and only if $\mathcal{R}_0(0)=R_0>1$. If $0<x_6<1$ then $\mathcal{E}_6$ exists if and only if $\mathcal{R}_0(x_6)>1$.

Dynamical regimes

The Jacobian matrix of the system of differential equations (2), in the main text, is given by

$$\mathbf{J}(S,I,x)=\begin{pmatrix}-\beta\,I-\mu & -\beta\,S & -\mu\,e\\ \beta\,I & \beta\,S-(\mu+\gamma) & 0\\ 0 & \mathbf{J}_{2,3}(I,x) & \mathbf{J}_{3,3}(I,x)\end{pmatrix}\tag{6}$$

where

$$\mathbf{J}_{2,3}(I,x)=\kappa\,x\,(1-x)\,\left(P^{(I)}(\pi_{V|N}(I,x))-P^{(I)}(\pi_{N|V}(I,x))\right)$$

and

$$\mathbf{J}_{3,3}(I,x)=\kappa\,x\,(1-x)\,\left(P^{(x)}(\pi_{V|N}(I,x))-P^{(x)}(\pi_{N|V}(I,x))\right)+\kappa\,(1-2x)\,\left(P(\pi_{V|N}(I,x))-P(\pi_{N|V}(I,x))\right)$$

The derivatives are given by

$$\begin{aligned}P^{(I)}(\pi_{V|N}(I,x)) &= cP(\pi_{V|N}(I,x))(1-P(\pi_{V|N}(I,x)))\left[-\sum_{j=0}^{3}p_j\,\omega_N'((I+\varepsilon)\,p_j)\,u_{N,o}(J_j-B)+\right.\\ &\left.\sum_{j=0}^{3}(1-e)\,p_j\,\omega_N'((1-e)\,(I+\varepsilon)\,p_j)\,u_{N,o}(J_j-B-m)\right]\\ P^{(I)}(\pi_{N|V}(I,x)) &= -c\,P(\pi_{N|V}(I,x))(1-P(\pi_{N|V}(I,x)))\left[-\sum_{j=0}^{3}p_j\,\omega_V'((I+\varepsilon)\,p_j)\,u_{V,o}(J_j-B)+\right.\\ &\left.\sum_{j=0}^{3}(1-e)\,p_j\,\omega_V'((1-e)\,(I+\varepsilon)\,p_j)\,u_{V,o}(J_j-B-m)\right]\end{aligned}$$

and

$$
\begin{aligned}
P^{(x)}(\pi_{V|N}(I,x)) &= c\,(\delta_V+\delta_N)\,P(\pi_{V|N}(I,x))(1-P(\pi_{V|N}(I,x)))\\
P^{(x)}(\pi_{N|V}(I,x)) &= -c\,(\delta_V+\delta_N)\,P(\pi_{N|V}(I,x))(1-P(\pi_{N|V}(I,x)))
\end{aligned}
$$

Equilibrium point $\mathcal{E}_1$ (pure vaccinator, disease-free) is stable if the disease is not highly contagious and the vaccine is not very scary compared to the strength of social norms, exactly when $\mathcal{R}_0(1) < 1$ (or $e > 1 - \dfrac{1}{R_0}$) and $\delta_V > \mathcal{M}(0)$. In contrast, $\mathcal{E}_2$ (non-vaccinator, disease-free) is stable when $\mathcal{R}_0(0) = R_0 < 1$ and $\mathcal{M}(0) > -\delta_N$. The equilibrium point $\mathcal{E}_3$ (partial vaccinator, disease-free) is not stable whenever it exists (since $\mathbf{J}_{3,3}(0, x_3) > 0$): for this equilibrium, social norms will always tend to move vaccine coverage up or down, hence the steady state is unstable. Equilibrium point $\mathcal{E}_4$ (pure vaccinator, disease-endemic) is stable if and only if vaccinator pressure is sufficiently large, $\delta_V > \mathcal{M}(\frac{\mu}{\mu+\gamma}\,\frac{\mathcal{R}_0(1)-1}{R_0})$, and if $\mathcal{R}_0(1)$ falls within a particular range:

$$
\frac{2}{1+\dfrac{\sqrt{\gamma}}{\sqrt{\mu+\gamma}}} < \mathcal{R}_0(1) < \frac{2}{1-\dfrac{\sqrt{\gamma}}{\sqrt{\mu+\gamma}}} \tag{7}
$$

Notice that, the left hand side term of inequality (7) is strictly greater than 1 (ensuring existence of the equilibrium point). The two inequalities in (7) can be rewritten in terms of vaccine efficacy (e) as

$$
1-\frac{2\,(\mu+\gamma)+2\,\sqrt{\gamma\,(\mu+\gamma)}}{\mu\,R_0} < e < 1-\frac{2\,(\mu+\gamma)-2\,\sqrt{\gamma\,(\mu+\gamma)}}{\mu\,R_0} \tag{8}
$$

The right hand side term is strictly less than $1-\dfrac{1}{R_0}$ (exactly by $\dfrac{\gamma+(\sqrt{\gamma}-\sqrt{\mu})^2}{\mu\,R_0}$). This region will appear in the simulation as a narrow gap between the two limits, see Figure 2 (a) in the main text.

Equilibrium point $\mathcal{E}_5$ (no vaccinator, disease-endemic) is stable if and only if non-vaccinator pressure is sufficiently large, $\mathcal{M}(\frac{\mu}{\mu+\gamma}\,\frac{\mathcal{R}_0(0)-1}{R_0}) > -\delta_N$ and if $\mathcal{R}_0(0)$ falls within a certain range:

$$
\frac{2}{1+\dfrac{\sqrt{\gamma}}{\sqrt{\mu+\gamma}}} < \mathcal{R}_0(0) < \frac{2}{1-\dfrac{\sqrt{\gamma}}{\sqrt{\mu+\gamma}}} \tag{9}
$$

Again, the left hand side term is strictly greater than one and the right hand side term is very large that the upper inequality includes all of the the basic reproduction numbers of non-chronic childhood diseases.

(Note that $\dfrac{\sqrt{\gamma}}{\sqrt{\mu+\gamma}}$ is very close to one since lengths of incubation period of non-chronic childhood diseases is much shorter than average human life length.)

Finally, equilibrium point $\mathcal{E}_6$ (partial vaccinator, disease-endemic) is stable if

$$\delta_N + \delta_V < -\frac{\mu\, e}{\mu+\gamma}\,\mathcal{M}'(\frac{\mu}{\mu+\gamma}\,\frac{\mathcal{R}_0(x_6)-1}{R_0})$$

where $\mathcal{M}'$ is the derivative of $\mathcal{M}$, and if R_0 is sufficiently large,

$$\mathcal{R}_0(x_6) > \frac{2\kappa c(\delta_N+\delta_V)}{\mu} x_6(1-x_6)P_6(1-P_6)$$

where $P_6 = P\left(\pi_{V|N}(\frac{\mu}{\mu+\gamma}\,\frac{\mathcal{R}_0(x_6)-1}{R_0}, x_6)\right)$.

Appendix III: Cumulative prospect theory

Cumulative prospect theory (CPT) amends the violation of stochastic dominance axiom by the prospect theory; c.f. [7]. It can also incorporate a multiple gains and losses [4]. In CPT, weights are given in terms of cumulative probabilities and not the probabilities. First, $\Theta = (z_1, p_1; z_2, p_2; \dots; z_k, p_k)$ is split into gains and losses say $\Theta^+ = (0, 1-(q_1+\cdots+q_n); x_1, q_1; x_2, q_2; \dots; x_n, q_n)$ and $\Theta^- = (y_m, r_m; \dots; y_2, r_2; y_1, r_1; 0, 1-(r_1+\cdots+r_m))$ such that $0 < x_1 < x_2 < \cdots < x_n$ and $y_m < \cdots < y_2 < y_1 < 0$ where $n+m=k$. The rank-dependent utility is given by

$$\pi(\Theta) = \sum_{i=1}^{n}[\omega^+(\sum_{l=i}^{n} q_l) - \omega^+(\sum_{l=i+1}^{n} q_l)]\, u(x_i) + \sum_{j=1}^{m}[\omega^-(\sum_{l=j}^{m} r_l) - \omega^-(\sum_{l=j+1}^{m} r_l)]\, u(y_j)$$

with the convention that $\sum_{l=k+1}^{k} a_l = 0$. The weighting functions ω^+ and ω^- have the same form as ω (see equation (1) in the main text) but need not to have the same parameters' value. The new definition of utility works very well with the anomaly case in the PT of a prospect of only two possible outcomes with the same sign.

In the model of vaccine acceptance, all the prospect's values are negative; and the prospect is of the kind $\Theta^- = (y_m, r_m; \dots; y_2, r_2; y_1, r_1)$. Thus, $\pi(\Theta^-) = \sum_{j=1}^{m}[\omega^-(\sum_{l=j}^{m} r_l) - \omega^-(\sum_{l=j+1}^{m} r_l)]\, u(y_j)$. We use an approximation for $\omega^-(\sum_{l=j}^{m} r_l) - \omega^-(\sum_{l=j+1}^{m} r_l)$ that boils down to the form of the weight function of the PT, $\omega := \omega^-$, since $\omega^-(r_j) \simeq \omega^-(\sum_{l=j}^{m} r_l) - \omega^-(\sum_{l=j+1}^{m} r_l)$ for $j = 1, \dots, m-1$ as all $r_1, \dots, r_{m-1}$

are small and $\omega^-(\sum_{l=m}^m r_l) - \omega^-(\sum_{l=m+1}^m r_l) = \omega^-(r_m)$.

Appendix IV: The effect of the cognitive parameters

To understand the effect of the cognitive parameters at different levels of vaccine cost on vaccine acceptance rates and disease eradication, we split them into three groups: the utility function exponents α_V and α_N; the loss aversion indexes $\lambda_{V,c}$, $\lambda_{V,o}$, $\lambda_{N,c}$ and $\lambda_{N,o}$; and the weighting parameters η_V and η_N. We discuss each group separately and set the parameters of the other two groups equal to one. At vaccine efficacy $e = .95$ and for all the three groups, the more the vaccine is to cost, the larger the vaccinators' group pressure is needed to have he same stability region that secures the possibility of full vaccine acceptance on condition of large initial acceptance. Moreover, the disease will be eradicated at that vaccine efficacy (Figures A1, A2 and A3). Compare those figures to Figures A4, A5 and A6 illustrated for vaccine efficacy $e = .9$. In the later cases, the full vaccine acceptance without diseases eradication $\mathcal{E}_4$ appears in the place of $\mathcal{E}_1$ with slightly smaller group pressure needed to cover the same region in the parameter plane. Cognitive parameters, seemingly, at the fixed vaccintors' group pressure can lead to full vaccine refusal (Figures A1 (a), A2 (a) and A3 (a)) which warrant a careful consideration of subsidies and rewards when dealing with human perception to gain, loss and weighting of probabilities. As the cost of vaccination increases, a larger group pressure is required to maintain the same area of bistability in Figures A1 (a), A2 (a), A3 (a), A4 (a), A5 (a) and A6 (a), compare panel (b) in Figures A1, A2, and A3 to their respective panels (c) and (d) as well as in Figures A4, A5, and A6. In that case a large enough initial vaccine acceptance is required to reach a full vaccine acceptance and eradicate the disease.

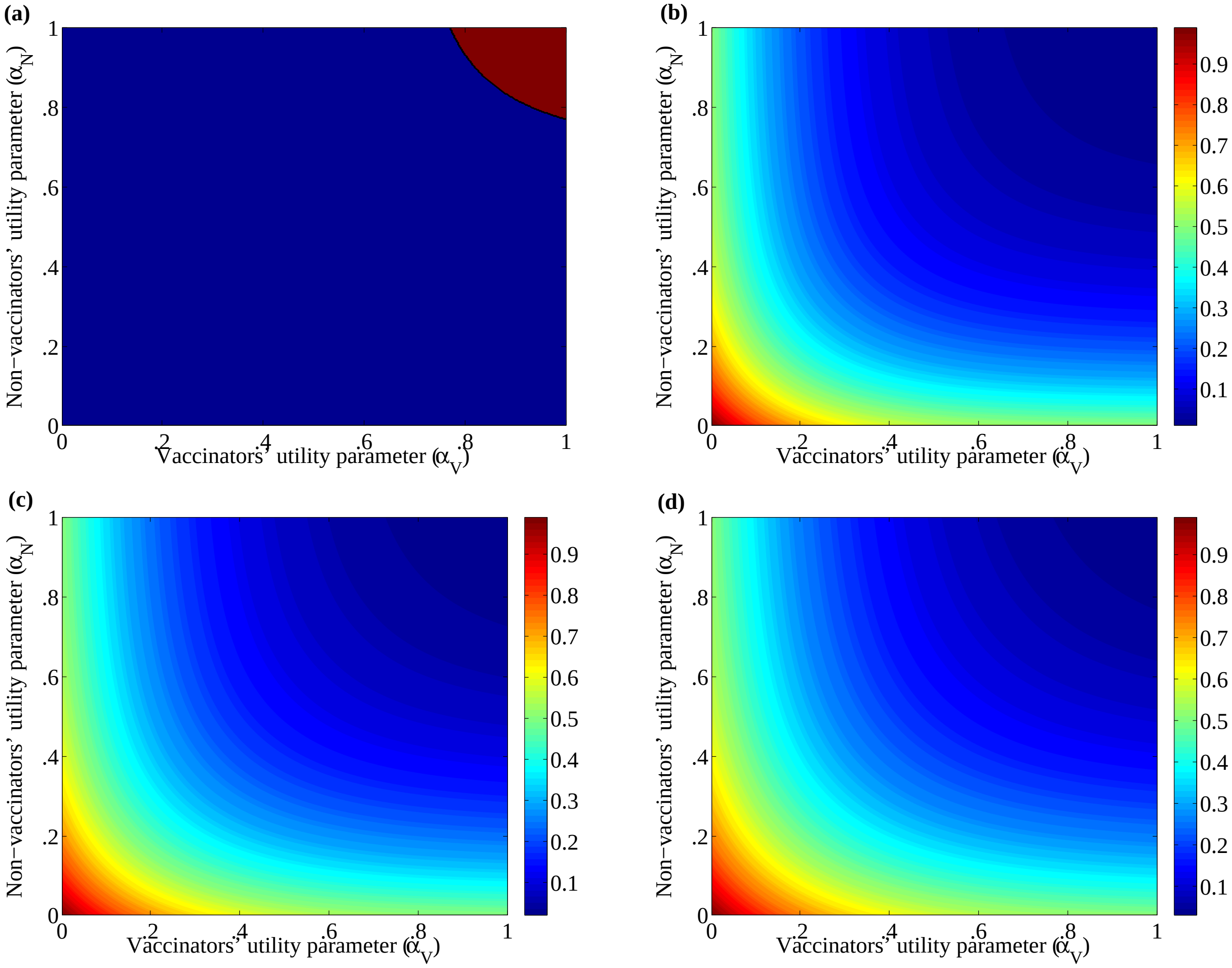

Figure A1. (a) α_V–α_N plane at $m = 0$ and $\delta_V = 0.02$. The equilibrium points $\mathcal{E}_1$ (pure vaccinator, disease-free) and $\mathcal{E}_5$ (no vaccinator, disease-endemic) are stable in the red region which includes the point $(1, 1)$ – corresponding to the rational decision model, given the values of the rest of the cognitive parameters below – whereas in the blue region the only stable point is $\mathcal{E}_5$. Contour plots for values of $\mathcal{M}(0)$ calculated at each pair of (α_V, α_N) at (b) $m = 0$, (c) $m = .01$, and (d) $m = .02$. The full vaccination equilibrium point $\mathcal{E}_1$ is stable in the regions where δ_V is larger than the value of $\mathcal{M}(0)$. Thus, (a) follows from (b) at $\delta_V = 0.02$. A similar contour plot of $\mathcal{M}(\frac{\mu}{\mu+\gamma}\ \frac{\mathcal{R}_0(0)-1}{R_0})$ (not shown here) shows that it is always positive so $\mathcal{E}_5$ is always stable. The rest of the parameters are $\kappa = 1.69, c = 1.46, e = .95, \eta_V = 1, \eta_N = 1, \lambda_{V,c} = \lambda_{V,o} = 1, \lambda_{N,c} = \lambda_{N,o} = 1$.

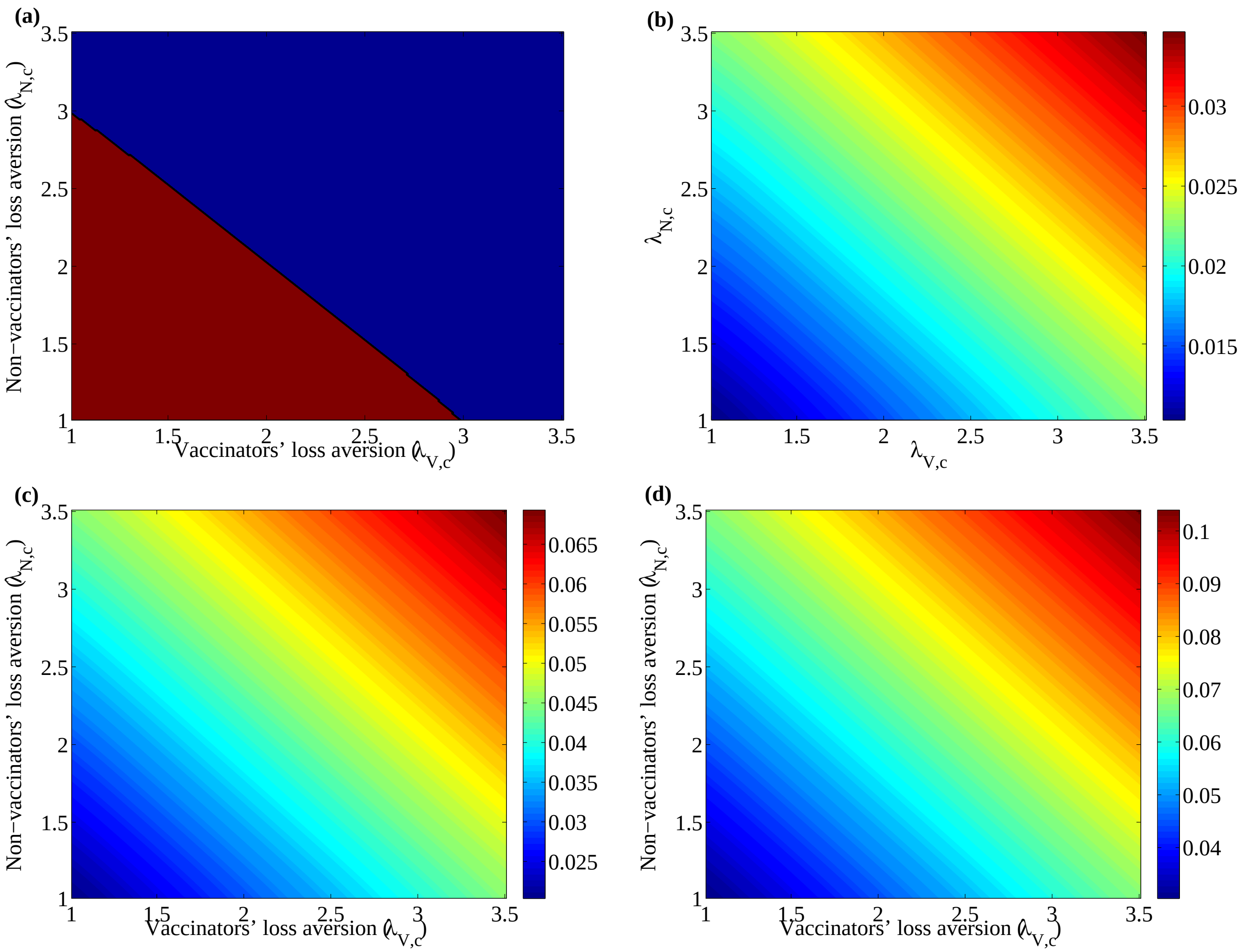

Figure A2. (a) $\lambda_{V,c}$–$\lambda_{N,c}$ plane with $\lambda_{V,o} = \lambda_{V,c}$ and $\lambda_{N,o} = \lambda_{N,c}$ at $m = 0$ and $\delta_V = 0.02$. The equilibrium points $\mathcal{E}_1$ (pure vaccinator, disease-free) and $\mathcal{E}_5$ (no vaccinator, disease-endemic) are stable in the red region which includes the point $(1, 1)$ – corresponding to the rational decision model, given the values of the rest of the cognitive parameters below – whereas in the blue region the only stable point is $\mathcal{E}_5$. Contour plots for values of $\mathcal{M}(0)$ calculated at each pair of $(\lambda_{V,c}, \lambda_{N,c})$ with $\lambda_{V,o} = \lambda_{V,c}$ and $\lambda_{N,o} = \lambda_{N,c}$ at (b) $m = 0$, (c) $m = .01$, and (d) $m = .02$. The full-vaccination equilibrium point $\mathcal{E}_1$ is stable in the regions where δ_V is larger than the value of $\mathcal{M}(0)$. Thus, (a) follows from (b) at $\delta_V = 0.02$. A similar contour plot of $\mathcal{M}(\frac{\mu}{\mu+\gamma} \frac{\mathcal{R}_0(0)-1}{R_0})$ (not shown here) shows that it is always positive so $\mathcal{E}_5$ is always stable. The rest of the parameters are
$\kappa = 1.69, c = 1.46, e = .95, \alpha_V = 1, \alpha_N = 1, \eta_V = 1, \eta_N = 1.$

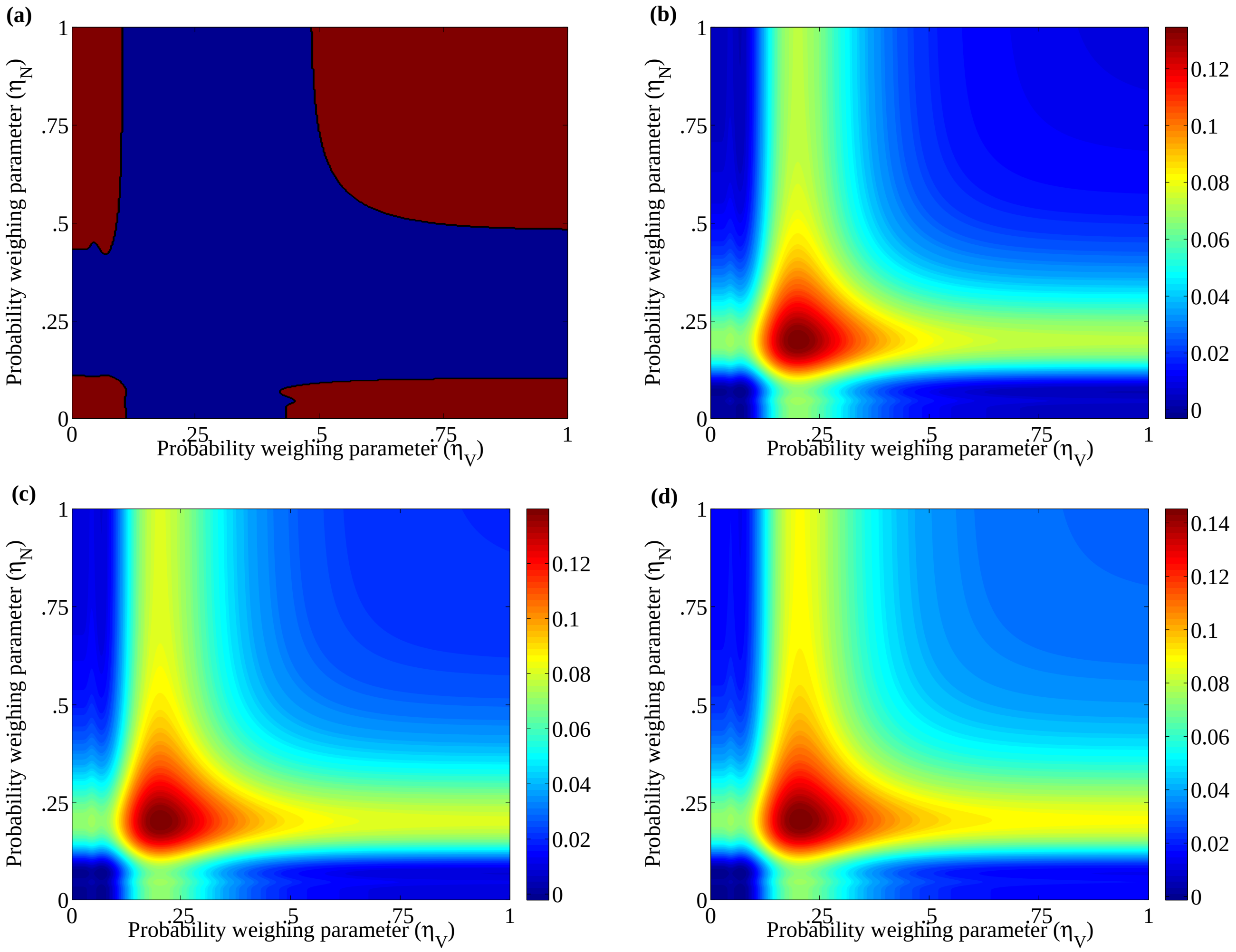

Figure A3. (a) η_V–η_N plane at $m = 0$ and $\delta_V = 0.02$. The equilibrium points $\mathcal{E}_1$ (pure vaccinator, disease-free) and $\mathcal{E}_5$ (no vaccinator, disease-endemic) are stable in the red region which includes the point $(1, 1)$ – corresponding to the rational decision model, given the values of the rest of the cognitive parameters below – whereas in the blue region the only stable point is $\mathcal{E}_5$. Contour plot for values of $\mathcal{M}(0)$ calculated at each pair of (η_V, η_N) at (b) $m = 0$, (c) $m = .01$, and (d) $m = .02$. The full vaccination equilibrium point $\mathcal{E}_1$ is stable in the regions where δ_V is larger than the value of $\mathcal{M}(0)$. Thus, (a) follows from (b) at $\delta_V = 0.02$. A similar contour plot of $\mathcal{M}(\frac{\mu}{\mu+\gamma}\,\frac{\mathcal{R}_0(0)-1}{R_0})$ (not shown here) shows that it is always positive so $\mathcal{E}_5$ is always stable. The rest of the parameters are $\kappa = 1.69, c = 1.46, e = .95, \alpha_V = 1, \alpha_N = 1, \lambda_{V,c} = \lambda_{V,o} = 1, \lambda_{N,c} = \lambda_{N,o} = 1$.

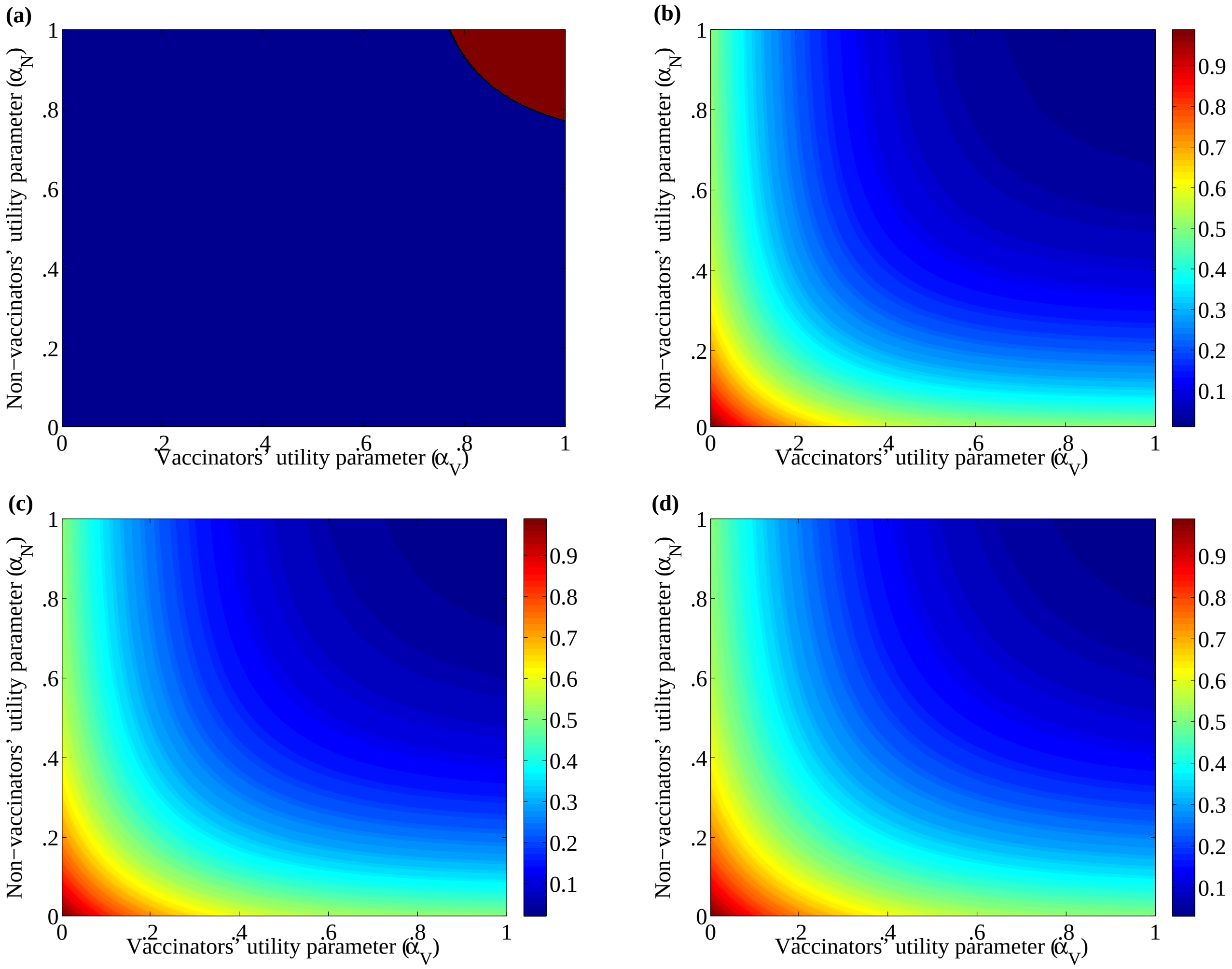

Figure A4. (a) α_V–α_N plane at $m = 0$ and $\delta_V = 0.02$. The equilibrium points $\mathcal{E}_4$ (pure vaccinator, disease-endemic) and $\mathcal{E}_5$ (no vaccinator, disease-endemic) are stable in the red region which includes the point $(1, 1)$ – corresponding to the rational decision model, given the values of the rest of the cognitive parameters below – whereas in the blue region the only stable point is $\mathcal{E}_5$. Contour plots for values of $\mathcal{M}(\frac{\mu}{\mu+\gamma}\,\frac{\mathcal{R}_0(1)-1}{R_0})$ calculated at each pair of (α_V, α_N) at (b) $m = 0$, (c) $m = .01$, and (d) $m = .02$. The full vaccination equilibrium point $\mathcal{E}_4$ is stable in the regions where δ_V is larger than the value of $\mathcal{M}(\frac{\mu}{\mu+\gamma}\,\frac{\mathcal{R}_0(1)-1}{R_0})$. Thus, (a) follows from (b) at $\delta_V = 0.02$. A similar contour plot of $\mathcal{M}(\frac{\mu}{\mu+\gamma}\,\frac{\mathcal{R}_0(0)-1}{R_0})$ (not shown here) shows that it is always positive so $\mathcal{E}_5$ is always stable. The rest of the parameters are $\kappa = 1.69, c = 1.46, e = .9, \eta_V = 1, \eta_N = 1, \lambda_{V,c} = \lambda_{V,o} = 1, \lambda_{N,c} = \lambda_{N,o} = 1$.

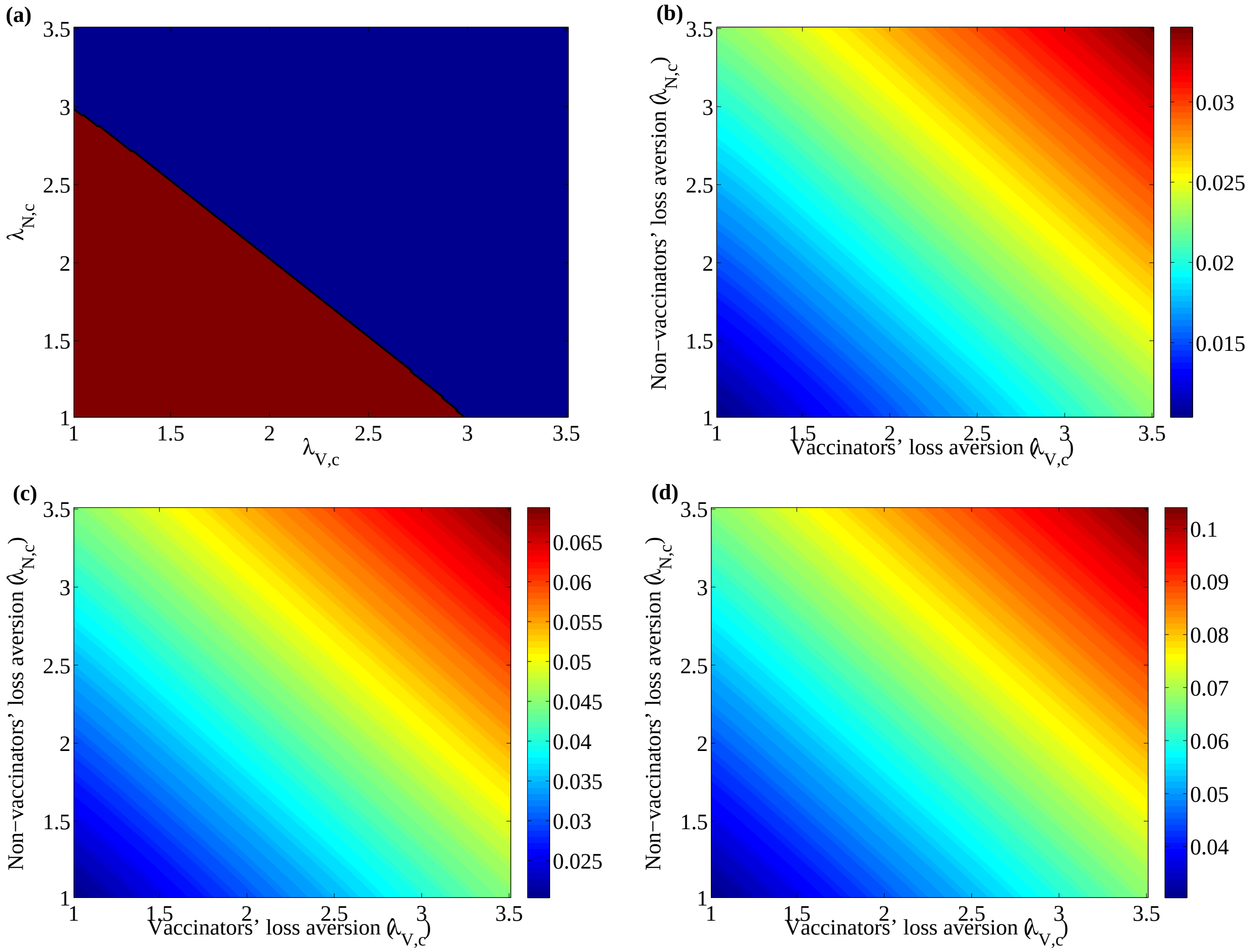

Figure A5. (a) $\lambda_{V,c}$–$\lambda_{N,c}$ plane with $\lambda_{V,o} = \lambda_{V,c}$ and $\lambda_{N,o} = \lambda_{N,c}$ at $m = 0$ and $\delta_V = 0.02$. The equilibrium points $\mathcal{E}_4$ (pure vaccinator, disease-endemic) and $\mathcal{E}_5$ (no vaccinator, disease-endemic) are stable in the red region which includes the point $(1, 1)$ – corresponding to the rational decision model, given the values of the rest of the cognitive parameters below – whereas in the blue region the only stable point is $\mathcal{E}_5$. Contour plots for values of $\mathcal{M}(\frac{\mu}{\mu+\gamma}\frac{\mathcal{R}_0(1)-1}{R_0})$ calculated at each pair of $(\lambda_{V,c}, \lambda_{N,c})$ with $\lambda_{V,o} = \lambda_{V,c}$ and $\lambda_{N,o} = \lambda_{N,c}$ at (b) $m = 0$, (c) $m = .01$, and (d) $m = .02$. The full-vaccination equilibrium point $\mathcal{E}_4$ is stable in the regions where δ_V is larger than the value of $\mathcal{M}(\frac{\mu}{\mu+\gamma}\frac{\mathcal{R}_0(1)-1}{R_0})$. Thus, (a) follows from (b) at $\delta_V = 0.02$. A similar contour plot of $\mathcal{M}(\frac{\mu}{\mu+\gamma}\frac{\mathcal{R}_0(0)-1}{R_0})$ (not shown here) shows that it is always positive so $\mathcal{E}_5$ is always stable. The rest of the parameters are $\kappa = 1.69, c = 1.46, e = .9, \alpha_V = 1, \alpha_N = 1, \eta_V = 1, \eta_N = 1$.

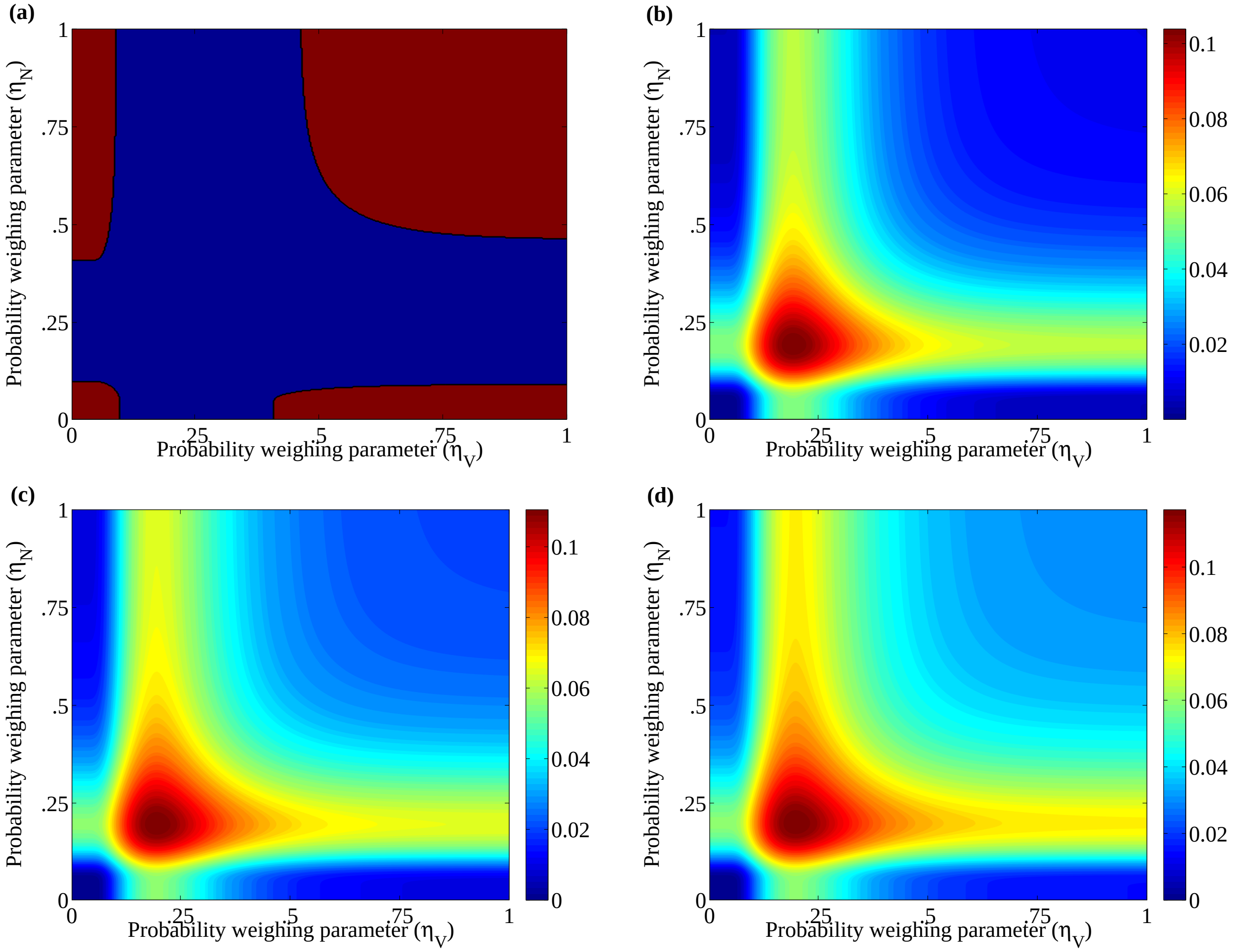

Figure A6. (a) η_V–η_N plane at $m = 0$ and $\delta_V = 0.02$. The equilibrium points $\mathcal{E}_4$ (pure vaccinator, disease-endemic) and $\mathcal{E}_5$ (no vaccinator, disease-endemic) are stable in the red region which includes the point $(1, 1)$ – corresponding to the rational decision model, given the values of the rest of the cognitive parameters below – whereas in the blue region the only stable point is $\mathcal{E}_5$. Contour plot for values of $\mathcal{M}(\frac{\mu}{\mu+\gamma}\,\frac{\mathcal{R}_0(1)-1}{R_0})$ calculated at each pair of (η_V, η_N) at (b) $m = 0$, (c) $m = .01$, and (d) $m = .02$. The full vaccination equilibrium point $\mathcal{E}_4$ is stable in the regions where δ_V is larger than the value of $\mathcal{M}(\frac{\mu}{\mu+\gamma}\,\frac{\mathcal{R}_0(1)-1}{R_0})$. Thus, (a) follows from (b) at $\delta_V = 0.02$. A similar contour plot of $\mathcal{M}(\frac{\mu}{\mu+\gamma}\,\frac{\mathcal{R}_0(0)-1}{R_0})$ (not shown here) shows that it is always positive so $\mathcal{E}_5$ is always stable. The rest of the parameters are $\kappa = 1.69, c = 1.46, e = .9, \alpha_V = 1, \alpha_N = 1, \lambda_{V,c} = \lambda_{V,o} = 1, \lambda_{N,c} = \lambda_{N,o} = 1$.

References

1. Centers for Disease Control and Prevention, Vaccines: Vac-Gen/Some Misconceptions - Risk from disease versus risk from vaccines. (2011) Available at: http://www.cdc.gov/vaccines/vac-

gen/6mishome.htm. (Accessed: 15th March 2014)

2. Bauch, C. T. & Bhattacharyya, S. Evolutionary game theory and social learning can determine how vaccine scares unfold. *PLoS Comput. Biol.* **8,** e1002452 (2012).

3. Trepel, C., Fox, C. R. & Poldrack, R. A. Prospect theory on the brain? Toward a cognitive neuroscience of decision under risk. *Cognitive Brain Res.* **23,** 34–50 (2005).

4. Tversky, A. & Kahneman, D. Advances in prospect theory: Cumulative representation of uncertainty. *J. Risk Uncertainty* **5,** 297–323 (1992).

5. Wu, G. & Gonzalez, R. Curvature of the probability weighting function. *Manage. Sci.* **42,** 1676–1690 (1996).

6. Oraby, T., Thampi, V. & Bauch, C. T. The influence of social norms on the dynamics of vaccinating behaviour for paediatric infectious diseases. *Proc. R. Soc. B: Behaviour* **281,** 20133172 (2014).

7. Fennema, H. & Wakker, P. P. Original and cumulative prospect theory: A discussion of empirical differences. *J. Behav. Decis. Making* **10,** 53–64 (1997).
